# A network approach for researching partnerships in health

**DOI:** 10.1186/1743-8462-2-22

**Published:** 2005-10-07

**Authors:** Jenny M Lewis

**Affiliations:** 1 Department of Political Science, University of Melbourne, Parkville, 3010, Australia

## Abstract

**Background:**

The last decade has witnessed a significant move towards new modes of governing that are based on coordination and collaboration. In particular, local level partnerships have been widely introduced around the world. There are few comprehensive approaches for researching the effects of these partnerships. The aim of this paper is to outline a network approach that combines structure and agency based explanations to research partnerships in health. Network research based on two Primary Care Partnerships (PCPs) in Victoria is used to demonstrate the utility of this approach. The paper examines multiple types of ties between people (structure), and the use and value of relationships to partners (agency), using interviews with the people involved in two PCPs – one in metropolitan Melbourne and one in a rural area.

**Results:**

Network maps of ties based on work, strategic information and policy advice, show that there are many strong connections in both PCPs. Not surprisingly, PCP staff are central and highly connected. Of more interest are the ties that are dependent on these dedicated partnership staff, as they reveal which actors become weakly linked or disconnected without them. Network measures indicate that work ties are the most dispersed and strategic information ties are the most concentrated around fewer people. Divisions of general practice are weakly linked, while local government officials and Department of Human Services (DHS) regional staff appear to play important bridging roles. Finally, the relationships between partners have changed and improved, and most of those interviewed value their new or improved links with partners.

**Conclusion:**

Improving service coordination and health promotion planning requires engaging people and building strong relationships. Mapping ties is a useful means for assessing the strengths and weaknesses of partnerships, and network analysis indicates concentration and dispersion, the importance of particular individuals, and the points at which they will fragment. A narrative approach adds an assessment of whether the partnerships are being used and valued. The approach outlined here, which examines structure and agency as separate but related explanations, has much to offer in examining partnerships.

## Introduction

Much discussion of policy making and governing at the beginning of the 21^st ^Century, indicates a significant shift in the model of governance across many sectors, away from an emphasis on competition between agencies (markets), to a model of inter-agency coordination and collaboration (networks). In Australia, this general trend is spelt out in the recent 'Connecting Government' paper [1]. This reflects the increasingly complicated arrangements for organizing and delivering services, which cross horizontal (levels of government) and vertical (government, private and third sector agencies) boundaries, and are in part a legacy of the earlier managerial and market based modes of governing [2]. It is helpful to conceive of many organizational contexts as network-like in order to understand them, without claiming that they are necessarily "good".

Partly driven by this shift, but also indicating a desire to find better approaches, there has been an astonishing growth in public policies which embrace the concepts of partnerships, alliances, collaborations and networks. Health policy has been a part of this broader trend, and there is no shortage of discussions of a range of collaborative forms of governing. In particular, partnerships of many varieties have become a key means for governing a range of policy initiatives at the local level [3].

This paper does not intend to make a full explication of what the characteristics of these partnerships are, or how they are being used. Neither does it address the question of whether there is in fact a clear commitment to them as a new form of governance, or whether they are better than previous approaches. Instead it explores new theories and methods for examining partnerships in health. As the current emphasis on partnerships shows no signs of abating, health policy research is in need of new concepts, methodologies and techniques for establishing their positive and negative effects.

While partnerships range from the bottom-up, locally self generated and voluntary, to the top-down, centrally steered and government mandated arrangements, those of central interest here are the latter, and are considered to be 'managed networks' [4]. Partnerships in the public sector often reflect efforts to institutionalise the positive effects of networking (such as increasing diversity by involving a greater range of actors) by requiring organisations and programs to have more formal connections to each other [5]. Those in focus here are created by government and are centrally steered with specific deliverables and targets defined by the centre rather than the individual partnerships. They cover defined geographical areas and have dedicated network coordinators. Leadership is undertaken by formal agencies rather than by mobilised communities. They have some ability to shape their own local priorities, but within limits set by a central authority [4].

Evaluations of a number of these types of partnerships in the UK have demonstrated that there are benefits, but that partnerships also face substantial difficulties. It is a slow process which clashes with the demands of government for results in the short term, and the need for local partnerships to reflect national priorities [4]. The most ambitious of these was Health Action Zones, and a recent evaluation of this program proclaimed the need for a new body of theory about what these kinds of programs can reasonably be expected to deliver in the face of bewildering complexities [6]. This paper argues that it is not only theory that is needed but also more appropriate methods for exploring their pluses and minuses.

A number of partnership tools have been created. One of the most relevance here is VicHealth's Partnerships Analysis Tool [7], which encourages partners to examine the reason for the partnership, map their relationships, and complete a checklist on a number of features of the partnership. While the map of the partnership has a similar focus to what is of concern here – understanding relationships – it is based on people's views of different types of engagement at an organizational level. This approach is straightforward to apply but more detailed views of relationships between people are required to understand what is happening beyond structures, and in accounting for the agency of individuals.

This paper attempts to walk the line between structure and agency, by combining network mapping and analysis with narratives, both of which are based on the observations of individual participants in partnerships. Pure description explains nothing, yet reflects the complexity of reality, while abstract theorising and modelling explains much but only by ignoring the complexity of reality [8]. A seemingly fruitful way of examining both structure and agency stems from Gidden's structuration theory [9]. He argues that structures constrain and facilitate actions, and also bind actions so that patterns are generated and reproduced. In other words, people work from within a set of structural constraints and opportunities, but also create and sustain these structures through their actions.

While Gidden's approach is to examine structure and action in isolation, Jessop's strategic-relation approach goes beyond this to examine structure in relation to action and action in relation to structure [10]. He argues for combining structural and discursive approaches. This is what this paper attempts, by using an approach that examines social networks as a set of connections, as well as a narrative about those network connections.

A little explored set of concepts and analytical techniques useful for evaluating these partnerships is available from social network analysis, which focuses on analysing relational data. It encompasses tools for network visualisation and network analysis using graph theory, statistical and algebraic models [11], and a range of concepts aimed at examining global network structure, network sub-structures, and the position of individuals within these networks (see these and other books dealing with these methods: [11-13]). The second means for examining whether and how partnerships improve relationships, build trust and foster better collaboration and cooperation between agencies is to examine the use and value of relationships through narratives.

The aim of this paper is to outline a network approach for use in researching partnerships in health. In doing so, research based on Primary Care Partnerships in Victoria is used to illustrate how combining network concepts and methods with narratives can be used to answer important questions about partnerships. The approach used examines connections (ties) between people, through the use of network mapping and analysis, particularly looking at multiple ties between people. This provides information on whether there are connections between people, in relation to various purposes (structure), but uncovers little about how they use and value them (agency), which requires an exploration of the quality of relationships within partnerships. More information on this approach is contained in the methods section.

### Primary Care Partnerships

Primary Care Partnerships (PCPs) were introduced in Victoria in 2001. The stated aim of PCPs is to improve the health and well being of a catchment's population by better coordination of planning and service delivery. A second aim is to improve the experience of and outcomes for recipients and reduce the preventable use of hospital, medical and residential services [14].

The core agencies in PCPs are community health services, local governments, district nursing services, divisions of general practice, and aged care assessment services. In each locality, other agencies are also partners, based on local priorities. The initial PCP policy document emphasised consumer, carer and community involvement in the partnership [14]. In establishing PCPs, the Department essentially provided funding for each to employ a network coordinator, and project workers who took on roles that reflect the main priorities of service coordination and health promotion. In general, all PCPs began with a steering committee, and committees to deal with service coordination and health promotion. Each PCP has a chair, often drawn from one of the partner organisations.

The state health authority, the Department of Human Services (DHS) centrally steers these partnerships. The DHS central office role is one of policy direction and advice, while DHS regional offices are responsible for monitoring and accountability. Local governments are an important partner and PCPs usually cover two or three local government areas. Some 32 partnerships were established across the state initially, and each of them received an establishment grant from DHS on signing a partnership agreement.

### Methods

Combining considerations of structure and agency into one approach is no easy task. However, this is where network theories and methods provide great promise. To demonstrate the utility of social network analysis for examining partnerships, research on two of the original 32 (now 31) PCPs, is used to examine whether and how relationships between individuals and organizations changed and developed since the inception of these partnerships.

One of the PCPs is within the Melbourne metropolitan area, and one is in rural Victoria. The information presented is based on a survey of 19 people from the metropolitan PCP, and 18 from the rural PCP. Interviews in the rural PCP were conducted with the three people in the PCP office, nine of the 14 people from the steering committee, three DHS regional office personnel, and three members of the health promotion steering committee. Three of the people from the consumers and carers group were interviewed together but network information was not collected from them. Interviews in the metropolitan PCP were conducted with three people from the PCP office, 11 of the 15 people who were on the steering committee at the time, two DHS regional office personnel, and three members of the health promotion steering committee. This information is summarised in Table 1.

**Table 1:** Information on the two PCP surveys

	Number of interviewees	Composition of interviewees	Date of interviews
Metropolitan PCP	19	3 PCP 11/15 Steering Committee 2 DHS Regional Office 3 Health Promotion	Late 2002

Rural PCP	18	3 PCP 9/14 Steering Committee 3 DHS Regional Office 3 Health Promotion	Late 2002 to early 2003

Members of the partnerships were interviewed using face to face or telephone interviews, and all were recorded and then fully transcribed. Name generators (that is, asking people who they would contact in relation to something) are commonly used to collect network information based on a range of relationships (see [15,16] for examples). In network terms, people have multiple types of ties with each other. To capture these multiple relations, interviewees were asked the following:

1. Looking back over the last 6 months, who are the people you had the most contact with ***in order to do your work?***

2. Over the last 6 months, who did you go to most when you wanted to get ***strategic information about something in the PCP***?

3. Over the last 6 months, who did you go to most when you wanted to talk about ***policy in relation to this PCP or PCPs in general***?

No set number of nominations was required, and a set of prompts was used if people were having trouble with recall (the prompts were: in your agency; in your PCP; in DHS regional and central offices; and elsewhere). The names were written into a form by the interviewer during the interview and the tape recording was used to check names later if they had been missed during the interview. While the second and third questions gave similar lists of people (with a number of respondents saying that list was the same), not all people nominated the same set of others for both. So many interviewees made a clear distinction between strategic information and policy ties.

The interviews provide information on both network structure, in terms of three different types of ties, and on agency, as described by people within the partnerships. The information on network ties forms the basis of the network maps and analysis. More people were mentioned than appear in these diagrams (as interviewees were free to nominate whoever they chose), but only interviewees are included here. That is, a larger number of people were mentioned in both PCPs, but those named but not surveyed did not have the chance to nominate people in return. Network analysis relies on people being able to both be nominated and to nominate others in return, so only the 19 and 18 who were interviewed are included in the analysis. This does not mean that the others are non-respondents in the traditional sense, it simply reflects that networks in effect have no boundaries.

During the interviews, open-ended questions were used to gather narrative descriptions of relationships. Questions were centred around:

• involvement with people in other agencies before the PCP was established

• level of contact since its establishment

• whether and how relationships had changed because of the PCP.

The narratives from the transcriptions were simply grouped under these three headings.

## Results

### Network mapping

The visualisation of relationships generated by mapping network connections between participants provides a useful pictorial means for describing links between people. The figures that follow show the three types of ties (work, strategic information, policy advice) combined, with the thickness of the lines between nodes (people) reflecting the number of different types of ties. For the purposes of this mapping exercise, more different types of ties between people is taken to indicate a stronger relationship. The maps presented here are generated by Netdraw, which is part of the UCInet package [17]. Those people with the most ties are placed at the centre of the map by this software.

The different colours of the dots (people) reflect which organization a person is from, based on Table 2:

**Table 2:** 

Red	PCP office
Black	Local goverment
Blue	Hospital
Pink	Community health service
Green	DHS Regional office
White	Division of General Practice
Yellow	Other

**Figure 1:**
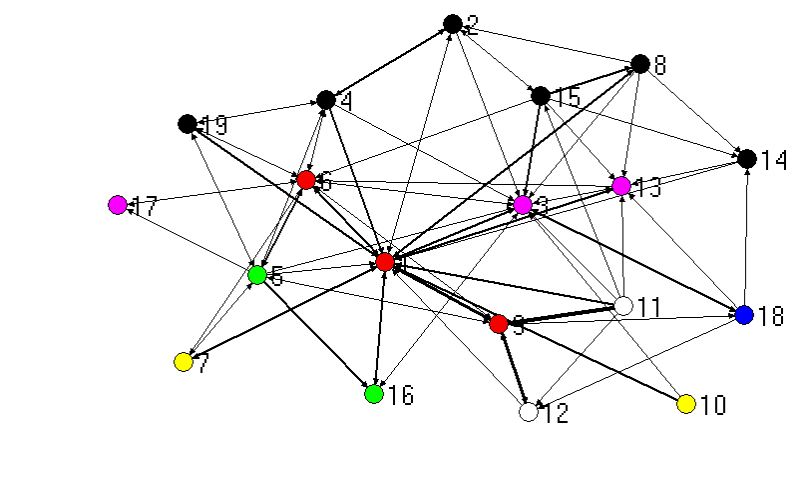
Network based on strength of ties for metropolitan PCP.

Figure 1 shows the ties for each of the 19 people interviewed in the metropolitan PCP, including the three PCP project staff. It shows a network with many strong connections, particularly to the PCP staff, and (unsurprisingly) centred around them. Community health service staff are also central, and this reflects that the chair of this PCP was located in a community health service. The ties between people are all based on different aspects of PCP engagement, so a point of interest here is how many of these ties are neither to or through PCP staff.

**Figure 2:**
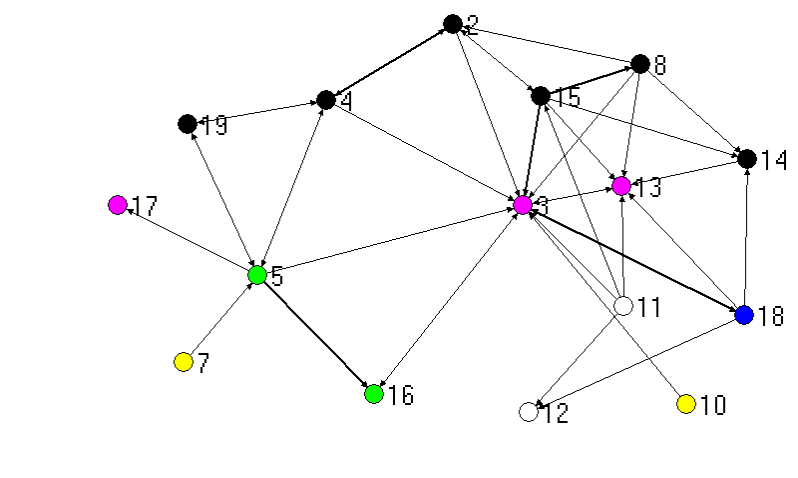
Network based on strength of ties for metropolitan PCP, without PCP staff.

Figure 2 shows the same network map with the three PCP staff excluded. The Chair of the PCP (3) becomes the most connected person once the PCP staff are removed, indicating the importance of this person and/or this organization in this locality. Without PCP staff, the remaining people are still connected into a single graph, but a number of them are now only weakly linked to others. Of interest is the divisions of general practice (11 and 12) which are especially weakly linked without the PCP in place.

**Figure 3:**
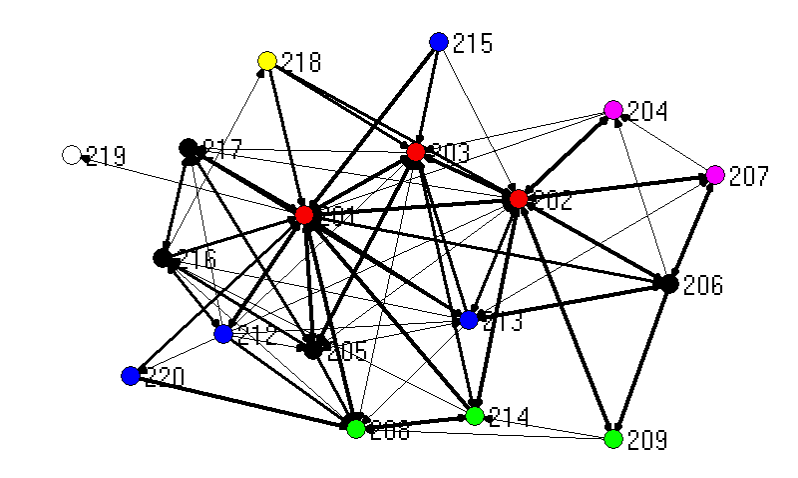
Network based on strength of ties for rural PCP.

**Figure 4:**
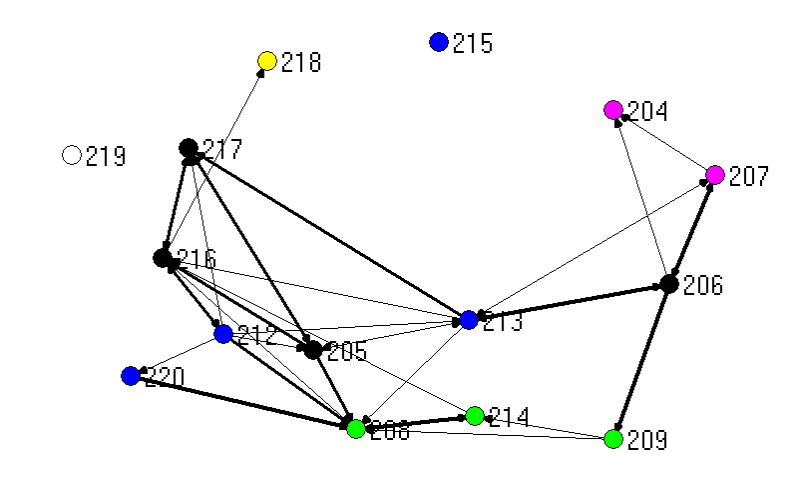
Network based on strength of ties for rural PCP, without PCP staff.

Figure 3 shows the ties for the 18 people surveyed in the rural PCP. Again, this network has many strong connections, particularly to the PCP staff, and is centred around them. Figure 4 is the same network minus the three PCP project staff. In contrast to the metropolitan PCP, the most connected person in this network, once the PCP staff have been removed, is located in the DHS regional office (208). Without the PCP staff, the rural PCP becomes disconnected, with two individuals isolated from the rest of the graph. These two people are from the division of general practice (219) and a hospital (215).

These figures illustrate the usefulness of examining ties between individuals across organisational boundaries in partnerships. It is not surprising that PCP staff are the most central in providing a connecting role to others, since this is after all what PCP staff are supposed to do. Removing the ties that directly involve PCP staff, indicates what a policy change to abolish PCPs would do, at least in the short term. Divisions of general practice provide an interesting case in point, as the maps show that they are likely to be the first to become disconnected or weakly connected without the impetus of the PCP. They are funded by the Commonwealth and have few financial incentives to get involved in state government policy initiatives, including through PCPs, so it is not surprising that they are weakly linked. This result concurs with an evaluation of PCPs across the state, that identifies these and other reasons why Divisions have low engagement in PCPs [18].

### Network measures

A large range of global network measures and network measures for individuals are available (see the growing literature on social network analysis for a more comprehensive guide). The following tables are based on the three different types of network ties discussed above – work, strategic information and policy advice. Table 3 contains overall network centralisation information. In-degree centrality is (in this case) a measure of the extent to which people choose others in relation to doing their work, gathering strategic information, or seeking advice about policy. Network centralization provides a measure of how concentrated all the ties are within a particular network, with a higher percentage of in-degree ties meaning that fewer people in a network have more of the ties directed to them, and a lower percentage indicating that the ties are more dispersed. The second measure – betweenness centrality – is an indication of the strategic importance of people within a network. A higher percentage means that fewer people provide bridging roles across the network, while a lower percentage means that more actors are playing this role.

**Table 3:** Centrality measures for networks (percentages)

PCP	Work	Strategic information	Policy advice
*Metropolitan*			
In-degree centrality	53.1	77.2	56.1
Betweenness centrality	28.4	6.0	27.8

*Rural*			
In-degree centrality	41.5	69.9	60.9
Betweenness centrality	21.2	21.3	15.6

Table 3 indicates that strategic information is the most centralised of these three types of networks, for both PCPs. This means that fewer people are sought out for this purpose than for policy advice, and work ties are the most dispersed of the three types. The pattern differs for betweenness centrality, with the measures being approximately equal across the three types in the rural PCP, but a much smaller percentage (and therefore many more people) playing a linking role for strategic information in the metropolitan PCP. This reinforces the earlier examination of the maps, which shows the fragmentation of the rural PCP without the PCP staff.

Table 4 includes individual measures of in-degree centrality and betweenness centrality for the three highest ranked people in each case. An individual's in-degree centrality score is the number of ties received, and so, a proxy measure for how important a person is seen to be by others in terms of informal resources possessed. Betweenness centrality is the number of non-redundant ties received. That is, it is the number of single ties that connect someone to others in the network. A high betweenness centrality (a high number of these ties) means that a person is in a position to act as a gatekeeper or bridge for information to flow throughout a network.

**Table 4:** Centrality measures for individuals within networks – highest ranked actors

PCP	Work	Strategic information	Policy advice
*Metropolitan*			
In-degree centrality	2 PCP staff PCP Chair	PCP CEO PCP Chair 1 DHS Region	2 PCP staff PCP Chair 1 DHS Region
Betweenness centrality	2 PCP staff PCP Chair	2 PCP staff PCP Chair	2 PCP staff Local Govt

*Rural*			
In-degree centrality	3 PCP staff	3 PCP staff 1 DHS Region	2 PCP staff 1 DHS Region
Betweenness centrality	2 PCP staff 1 Local Govt	2 PCP staff 1 DHS Region	2 PCP staff 1 DHS Region

A glance at Table 4 confirms the picture from the network maps that PCP staff are highly central in all three types of networks, and in both PCPs. In the metropolitan PCP, the Chair and one DHS regional staff member are also very central for strategic information and policy advice. In the rural PCP, one DHS regional office staff member is important for strategic information and policy advice. The betweenness centrality statistics show a similar picture, with PCP staff providing the bridges. Again the Chair in the metropolitan PCP is important, and a DHS regional officer is important in the rural case. An interesting addition is that local government staff appear to play bridging roles in both PCPs, although they are not central in terms of in-degree in either case.

Table 5 shows where the cut-points are for these three network types for both PCPs. A cut-point is a point which, if removed, causes the network to fragment. That is, it is a point at which a single, connected graph will become disconnected into two or more components. So cut-points indicate those people that are holding a network together. PCP staff, and particularly PCP CEOs feature in all three network types for the metropolitan PCP and two of the rural ones. The exception is policy advice in the rural PCP, where a DHS regional officer is the cut-point. Two local government officials in the metropolitan PCP are holding the policy advice network together, and a community health actor is important in the rural PCP in regard to work.

**Table 5:** Cut-points for individuals within networks

PCP	Work	Strategic information	Policy advice
*Metropolitan*			
	PCP CEO	PCP CEO PCP	PCP CEO PCP Chair 2 Local Govt

*Rural*			
	PCP CEO Comty Health	PCP CEO PCP	DHS Region

### Network narratives

Since the approach taken in this paper assumes that those involved in PCPs are not simply passive points in a network structure, but are actively creating and sustaining relationships with others in the PCP, this paper now considers how those involved in these PCPs use and value them.

Many interviewees in both PCPs argued that relationships between agencies, prior to the establishment of PCPs, had been adversely effected by an atmosphere of competition in their locality, reflecting the policy emphasis on purchaser-provider separation, compulsory competitive tendering and also local government amalgamations, under the previous state government. The following comment from the Chair in the metropolitan PCP (3), who is very central (see Figures 1 and 2), indicates how relationships were shaped by this:

"Relationships with other agencies were often strained. It was often the case that you were trying to work out what other agencies might be doing in terms of the tendering process."

A hospital person in the rural PCP (215), situated on the top edge of Figures 3 and 4 remarked on the previous lack of working together:

"In the pre-PCP environment we all conceptualised it, we all sat around and dreamed. That's as far as it got."

Everybody interviewed in these two PCPs thought they had greater engagement with other agencies than prior to the PCP, and almost all saw this as positive. The metropolitan PCP CEO (1), who is very central in Figures 1 and 2, commented:

"We've got people around the table who haven't been around the table before ... through the regular meetings and forums ... you can eye-ball someone and know who it is"

A less positive comment came from one (fairly peripheral) local government actor:

"I don't think that people have got time to waste ... I think it's just quite cumbersome to go to meeting after meeting and run into the same people" (14)

In the rural PCP, one community health actor (206) said:

"So networking is what it's all about and it really has opened a lot of doors as to who is out there, what they do and I guess getting them to recognise that they do have a health promotion role."

A telling comment on connections to the divisions of general practice, made by a DHS regional officer (208) was:

"I've probably dealt more with the Division of GPs in the last six months than I have in the previous five years and, even though they have a strong relationship with a few hospitals, there was never a need or a perceived need to talk to them directly but now that I've made the contacts I talk to them about lots of different things."

Finally, comments about the quality of relationships revealed that most people valued the partnerships and were using them for a variety of purposes. Several spoke of how trust had been built through the PCPs, leading to opportunities to do more things together. One of the weakly linked peripheral people (10 on figures 1 and 2) claimed:

"I think we're a lot better off, we're a lot better networked."

Another peripheral person (18) said:

"I think what happens now is there's more of a commitment and an understanding for working together rather than just knowing each other."

The division of general practice person in the rural PCP (219) argued:

"The PCP basically formalised that into a process with MOUs or contracts to in fact strengthen the ties or get people to have a better understanding of what was going on."

One centrally located local government person (205 in figures 3 and 4) said:

"I feel that the hospitals aren't as committed as the other agencies. The hospital management, I think they still see their primary role as, obviously, acute care and everything else is a tack on...."

The rural PCP Chair (217) said:

"I think it's about establishing networks. Because you know more people involved in different services, if something crosses your desk and it might be a funding application or whatever, if it rings bells you think, 'Oh yes I could talk to so and so about that, that links in to this program and we could do so and so together.' "

These comments add agency to the network structures discussed earlier, highlighting how relationships have changed and developed through the PCPs. Interestingly, a number of people were using networks as a concept in their comments and some spoke in quite specific network or partnership terms (overcoming boundaries and cut offs, more and better networking, developing relationships, working together, strengthening ties). Comments about the divisions of general practice and hospitals also resonate with their positions on the network maps (tending to be around the edges, as could be expected) and the network measures, which show they are not as central as local government, community health and DHS regional officials. They are the most likely to be disconnected or weakly linked without the intervention of PCP staff.

## Discussion and conclusion

Clear indications of how these partnerships were progressing in their first year can be drawn from this analysis. The aim of PCPs is to improve health and well being through better service coordination and health promotion planning. A first step towards this is engaging more people and building stronger relationships. Mapping reported ties provides a useful means for assessing structure and where the strengths and weaknesses of partnerships lie. Using network analysis techniques such as measuring centrality of different kinds and the points at which networks fragment, clearly highlights the overall concentration and dispersion of different types of networks, and the importance of particular individuals. And narrative descriptions of partnerships provide insights on agency – in this case, whether those involved are using and valuing the partnerships.

The focus in this paper is on evaluating new governance modes which could ultimately lead to better outcomes through a coordinated service where agencies understand each others roles and have ongoing relationships. This is intuitively better than one based on fragmentation, lack of information and few relationships, but the benefits need to be settled by empirical examination. The approach outlined here, based on analysing network structures and narratives, is useful for examining these changes. It is particularly valuable as a means for analysing linkages that should indicate increasing capacity in the early stages of such policies when clear improvements in outcomes might be some time off. But this approach cannot reveal whether outcomes have improved, and the intention of this paper is not to suggest that it can.

This paper has combined structural and agency-based explanations by collecting information on both and examining them as separate but related parts of the same puzzle. This introduction to some of the techniques available for collecting, visualising, and analysing network data, highlights their potential for researching partnerships. The toolbox of social network analysis is much bigger than this overview indicates, and the many and varied measurement techniques can be found in the growing literature on methods [11-13], in specialist journals (most notably *Social Networks*), and the websites of relevant associations, such as the International Network for Social Network Analysis [19]. Enthusiasm for these techniques needs to be tempered with considerations of agency. Conversely, a focus on narratives holds much appeal but tends to ignore structure. Both are required, as a reliance on either goes only half way to a strong approach to researching partnerships.

## Competing interests

The author(s) declare that they have no competing interests.
